# Supplementary material for: A Critical View of the Application of the APEX Software (Aqueous Photochemistry of Environmentally-Occurring Xenobiotics) to Predict Photoreaction Kinetics in Surface Freshwaters
Source: Molecules. 2019 Dec 18;25(1):9. doi: 10.3390/molecules25010009 (PMC7017383; doi:10.3390/molecules25010009)
Supplement: Supplementary file 1 [file molecules-25-00009-s001.zip › 3.2.4_gcc-4.4.0.zip!/3.2.4_gcc-4.4.0/3.2.4_gcc-4.4.0/doc/octave/html/interpreter/Plotting-Basics.html]

Plotting Basics - Untitled


Next: Advanced Plotting,
Up: Plotting


---

### 15.1 Plotting Basics

Octave makes it easy to create many different types of two- and
three-dimensional plots using a few high-level functions.

If you need finer control over graphics, see Advanced Plotting.

- Two-Dimensional Plots- Three-Dimensional Plotting- Plot Annotations- Multiple Plots on One Page- Multiple Plot Windows- Printing Plots- Interacting with plots- Test Plotting Functions
